# Machine Learning Versus Traditional Statistical Models for Emergency Department Volume Forecasting

**DOI:** 10.5811/westjem.62266

**Published:** 2026-10-14

**Authors:** Kian Huang, Enola Okonkwo, Haris Choudhary, Rahul Mhaskar, Nathan Lapaix, Michael Lozano, Reginald Saint-Hilaire

**Affiliations:** * University of South Florida, Morsani College of Medicine, Tampa, Florida; † University of South Florida, Morsani College of Medicine, Tampa General Hospital, Department of Emergency Medicine, Tampa, Florida; ‡ University of South Florida, Morsani College of Medicine, Division of Evidence-based Medicine and Outcomes Research, Tampa, Florida

## Abstract

**Introduction:**

Emergency department (ED) crowding harms patient care and strains hospital operations. Forecasting daily patient arrivals can help ED medical directors, department chairs, and hospital administrators plan staffing and bed capacity in advance, rather than reacting after a surge has already occurred. This study compared five computer-based forecasting methods, including three machine learning models (eXtreme gradient boosting [XGBoost], light gradient boosting machine [LGBM], and long short-term memory [LSTM]) and two traditional statistical models (autoregressive integrated moving average [ARIMA] and seasonal ARIMA [SARIMA]), to determine which most accurately predicts ED patient volume seven days in advance, using eight years of daily visit data from an academic freestanding ED.

**Methods:**

This retrospective study analyzed 3,202 consecutive days of daily patient volume (March 27, 2017, to December 31, 2025) from an academic freestanding ED with approximately 31,102 annual visits. Each model used the preceding 365 days of data to forecast the subsequent 7 days, repeated across the study period to reflect the weekly cycle by which most EDs plan staffing. Our primary measure of accuracy was mean absolute error (MAE), defined as the average number of patients per day by which a forecast missed the actual volume. Secondary measures included root mean squared error (RMSE), mean absolute percentage error (MAPE), the coefficient of determination (R^2^, a score from 0 to 1 reflecting how well predictions tracked actual day-to-day volume), and the percentage of forecasts accurate within 5, 10, 15, and 20 patients, thresholds selected for their relevance to staffing decisions. The best-performing model on initial metrics, XGBoost, served as the reference model for statistical comparison using the Diebold-Mariano (DM) test, which determines whether an observed difference in accuracy between two models is statistically meaningful or attributable to chance.

**Results:**

Among 453 rolling weekly forecast windows, SARIMA, XGBoost, and LGBM demonstrated the lowest absolute errors and best forecasting of patient volume, with MAE values of 9.60 (95% CI, 9.29–9.91), 9.61 (95% CI, 9.31–9.91), and 9.64 (95% CI, 9.33–9.94) patients/day, respectively; these three models therefore missed the actual daily patient count by approximately 9 to 10 patients, on average. Models ARIMA and LSTM were less accurate, with mean MAE values of 10.54 (95% CI, 10.21–10.87) and 12.01 (95% CI, 11.63–12.39) patients per day. Relative to the XGBoost reference (MAPE 13.82%), ARIMA (MAPE 14.93%) and LSTM (MAPE 17.27%) showed a marked decrease in predictive accuracy. For operational planning, the top-performing models (SARIMA, XGBoost, and LGBM) correctly predicted daily volume within a margin of 20 patients in 90.3% to 90.6% of instances, while LSTM performed less well and reached this threshold in only 83.3% of forecasts. Statistical testing confirmed these differences were unlikely due to chance, with XGBoost significantly outperforming ARIMA (DM = −6.09, *P* < .001) and LSTM (DM = −6.96, *P* < .001).

**Conclusion:**

Models SARIMA, XGBoost, and LGBM performed comparably well, and all three would provide reliable, operationally useful forecasts for ED staffing and resource allocation decisions. A traditional statistical method, SARIMA, which is simpler and less resource-intensive to implement, performed as well as the newer machine learning methods evaluated in this study. This finding suggests that hospitals do not necessarily need to invest in more complex artificial intelligence tools to obtain accurate ED volume forecasts.

## INTRODUCTION

Emergency departments (EDs) are essential access points for acute medical care, with over 150 million visits in the US in 2022 driven by both acute and chronic needs.^1^ Rising utilization strains ED resources and exacerbates crowding, leading to prolonged waits, delays in care, higher rates of patients leaving without being seen, and increased short-term mortality.^2^ Crowding also affects staff morale, operational efficiency, and healthcare costs.^2^ For ED medical directors, department chairs, and hospital administrators, these problems often translate into a practical question addressed on a weekly basis: how many physicians, nurses, and beds will be needed on a given day, and how can that number be predicted with sufficient lead time to act on it?

Hospitals traditionally rely on retrospective averages, calendar-based scheduling, and managerial judgment to align staffing with demand.^3^,^4^ These methods often fail to accommodate short-term surges influenced by seasonal illness, weather, or emergent events, leading to resource misallocation.^5^ Data-driven forecasting, in which a hospital’s own historical volume data are used to mathematically predict future patient counts, has therefore become increasingly important, although it remains unclear whether simpler, established statistical methods or newer, more computationally intensive methods perform best for this task.

One category of forecasting tool, known as time series models, learns from a hospital’s own historical patterns (eg, recognizing that Mondays are consistently busier than Tuesdays, or that volume rises predictably each winter) and projects these patterns forward. Two long-established time series methods are autoregressive integrated moving average (ARIMA) and its seasonal counterpart, seasonal ARIMA (SARIMA). Both are traditional statistical techniques, similar in principle to methods long used in economic and business forecasting; neither requires artificial intelligence (AI) or specialized computing infrastructure, and both can typically be implemented with standard statistical software. Schweigler et al showed SARIMA outperformed historical averages in forecasting ED occupancy across multiple hospitals.^4^ Gul et al reviewed ED forecasting methods and noted that while traditional statistical models, including linear regression and ARIMA, achieve mean absolute percentage errors (MAPE—the average forecast error expressed as a percentage of actual volume) under 15%, they often struggle with sudden, irregular fluctuations, suggesting a possible role for more complex approaches.^3^ More recently, Al Yarubi et al compared SARIMA with two additional statistical forecasting methods (Holt-Winters and Prophet) for predicting hourly ED crowding and found that each offered distinct strengths depending on the forecasting horizon, reinforcing that no single traditional method is universally superior.^6^

More recently, investigators have applied machine learning, a branch of AI in which a computer program identifies complex, often nonlinear patterns in data rather than following prespecified statistical rules, to ED forecasting. Three such methods evaluated in this study were eXtreme gradient boosting (XGBoost), light gradient boosting machine (LGBM), and long short-term memory (LSTM) networks. eXtreme gradient boosting and LGBM are gradient boosting algorithms, meaning they construct many simple decision rules in sequence, with each successive rule correcting the errors of those preceding it; this allows them to capture complex relationships between calendar patterns and patient volume that simpler statistical models may not detect.^7^

**Population Health Research Capsule**

What do we already know about this issue?

*Both traditional statistics and machine learning can forecast emergency department (ED) volume, but which approach performs best for staffing remains unclear.*

What was the research question?

*Do machine learning models forecast 7-day ED volume more accurately than autoregressive integrated moving and seasonal average?*

What was the major finding of the study?

*XGBoost had mean absolute error (MAE). 9.61 (95% CI, 9.31–9.91) vs LSTM MAE 12.01 (95% CI, 11.63–12.39) patients/day; Diebold-Mariano =* −*6.96, P < .001.*

How does this improve population health?

*A simple, low-cost statistical model forecasts ED volume as well as machine learning, expanding access to accurate staffing tools.*

The XGBoost method is known for its computational efficiency, ability to handle missing data, and strong performance in modeling these complex, nonlinear relationships.^8^ The LGBM method is a faster, more memory-efficient variant of the same gradient boosting approach that can accelerate training while maintaining comparable predictive accuracy.^8^ In prior ED forecasting studies, XGBoost models incorporating calendar and recent-history features have achieved error rates of approximately 5–14% (MAPE) and *R*^2^ values up to 0.81.^8^,^9^ Hu et al similarly demonstrated that incorporating real-time information improved short-term forecasts of ED arrivals compared with methods relying on historical averages alone.^10^ The LGBM method has also shown competitive results when paired with extensive feature engineering, which is the process of constructing informative input variables from raw data (described further in the methods section).^8^

The LSTM networks are a more computationally intensive form of deep learning, a subset of machine learning loosely modeled on neural connections in the brain, that processes sequential data by selectively retaining and discarding information over time through internal gating mechanisms, allowing the model to learn long-range dependencies such as multiweek trends.^11^ Cheng and Kuo demonstrated that LSTM reduced mean absolute error (MAE—the average forecast error in raw units) in predicting ED wait time by 9.7% compared with linear regression.^12^ Ming et al reported strong LSTM performance in daily ED arrival forecasting, achieving a MAPE of 4.87%.^13^

Given the persistent challenges of ED resource allocation and the growing promise of machine learning, this study evaluates XGBoost, LGBM, and LSTM, three machine learning approaches, against ARIMA and SARIMA, two established statistical benchmarks, in forecasting daily patient volume at an academic freestanding ED. None of these five methods are proprietary commercial products; all are publicly available, open-source statistical and machine learning techniques that can be implemented by a hospital’s data analytics team, biomedical informatics group, or a contracted data scientist with access to the ED’s own historical volume data and standard statistical programming skills; we used Python (Python Software Foundation). An ED medical director does not require personal expertise in AI to benefit from this work; rather, our objective is to inform decisions such as whether investment in more advanced, and typically more resource-intensive and expertise-intensive, forecasting tools is warranted, or whether simpler, well-established statistical methods already perform comparably well for operational planning. By systematically comparing these approaches on identical data, we aim to provide ED leaders and hospital administrators with evidence to guide such decisions.

## METHODS

### Study Design and Data

This retrospective modeling study used daily patient volume data from a 20-bed academic ED affiliated with a tertiary-care university hospital. The ED serves approximately 31,102 patients annually, with a diverse urban and suburban population and a typical acuity mix, making findings potentially generalizable to similar freestanding or hospital-based EDs. The dataset spanned March 27, 2017, to December 31, 2025, totaling 3,202 consecutive days. Because staffing and resource allocation decisions at most EDs, including ours, are typically made on a weekly basis, we designed our forecasting approach around 7-day (weekly) prediction windows to mirror this real-world planning cycle. The calendar date served as the temporal index from which all predictor variables (described below) were calculated. The outcome measure was daily ED census, defined as the total number of registered ED visits per calendar day.

Daily ED census data were imported and processed in Python 3.10. Data quality checks identified no missing dates or duplicate entries requiring correction. Interpolation and imputation procedures were prespecified but ultimately not applied, as the dataset was complete. No automated outlier removal was performed. The dataset encompasses the COVID-19 pandemic period (March 2020 onward), during which ED volumes experienced unprecedented disruptions.^14^ These fluctuations were retained to assess model performance under real-world operational conditions with external shocks, rather than presenting an artificially “clean” picture that would not reflect a real ED’s experience. A 365-day rolling training window was implemented to capture complete seasonal cycles. Specifically, at each step the models were provided a full year of recent history before being asked to forecast the following week, ensuring that annual patterns, such as winter respiratory illness surges, were consistently represented in the training data. At each iteration, models were trained using the preceding 365 days of data and generated a single 7-day forecast for the subsequent week, mimicking a weekly operational planning cycle. Weekly 7-day forecasts were selected to balance computational feasibility with prediction accuracy.

### Feature Engineering

Feature engineering refers to the process of transforming a raw calendar date into structured variables that a forecasting model can use to learn patterns in ED utilization. We constructed several categories of these input variables, informed by prior ED forecasting literature and domain expertise. No patient-level demographic or clinical variables were included; all predictors were derived from the date index and historical volume values.

Calendar-based categorical features (year, month, day of month, day of week, week of year, and calendar quarter) captured seasonal trends, operational cycles, and known weekday clustering in ED utilization, allowing the model to learn that Mondays or December differ systematically from other days or months. To prevent the model from incorrectly treating time as a simple linear trend, we applied sine and cosine transformations, a standard mathematical technique for representing repeating, cyclical phenomena (eg, December is calendrically proximate to January despite occupying opposite ends of a yearly count) to represent repeating monthly and weekly seasonality. Behavioral patterns were encoded through binary (yes/no) indicators for weekends and major US federal holidays.

Historical utilization patterns were captured through multiple lagged values, that is, the actual patient volume from 1, 3, 7, 14, 28, and 30 days earlier, allowing the models to learn short-term recency effects, weekly repetition, and longer seasonal recovery patterns. We also added smoothed trend variables using rolling (moving) averages over 3-, 7-, 14-, and 30-day windows, along with rolling standard deviations, rolling minimum/maximum values, and percentile-based features; each was calculated using only prior information to avoid temporal leakage, defined as inadvertently allowing a model to see future data it would not have had access to in real time. Additional variables captured momentum, such as 1-day and 7-day changes in volume, percentage-change ratios, exponentially weighted moving averages, and same-weekday memory variables (eg, volume on the same weekday in the prior week). Standardized (z-score–like) features quantified how far current volume deviated from recent rolling averages, helping models, particularly LSTM, learn to recognize unusual surges or unusually quiet days.

### Forecasting Framework and Model Training

The technical configuration details below are reported to allow other investigators to reproduce this study; readers primarily interested in the clinical and operational implications may wish to proceed to Evaluation Metrics and Results.

We evaluated five forecasting models: XGBoost, LGBM, LSTM, ARIMA, and SARIMA. For the gradient boosting models, XGBoost was configured with 75 estimators, learning rate 0.05, maximum depth 6, column subsample fraction 0.9, row subsample fraction 0.8, and L1/L2 regularization (α = 0.1, λ = 0.1). The LGBM was configured with 31 leaves per tree, learning rate 0.05, feature fraction 0.9, and bagging fraction 0.8 applied every five iterations. Both models used a fixed random seed for reproducibility. Hyperparameters were tuned once using an initial calibration period restricted to the earliest portion of the time series, preserving strict temporal ordering. No data from the rolling evaluation period were used during hyperparameter selection. Selected hyperparameters were fixed prior to walk-forward validation to prevent information leakage.

The LSTM model employed a two-layer recurrent neural network architecture consisting of a 64-unit LSTM layer followed by a 32-unit LSTM layer, both using dropout regularization of 0.2. The input sequence window was 30 days, with the model trained to forecast a 7-day horizon. Training was performed using a batch size of 64, with early stopping triggered after five consecutive epochs without improvement in validation loss. The model was implemented in TensorFlow/Keras version 2.20 (Google LLC) using the Adam optimizer and Huber loss with δ = 10.0 as the objective function.

### Evaluation Metrics

Model performance was assessed using two complementary approaches: aggregate metrics summarizing accuracy across all forecasts combined, and weekly-level metrics capturing how much a model’s accuracy varied from week to week, a measure of consistency referred to as error dispersion, meaning how spread out or variable a model’s daily errors are, rather than simply their average.

For aggregate performance, we calculated four accuracy measures across all rolling 7-day forecast windows. The MAE is the average number of patients per day a forecast missed by, regardless of whether the model over-predicted or under-predicted; this was our primary outcome measure. Root mean squared error (RMSE) is similar to MAE but gives additional weight to large misses, making it more sensitive to occasional large forecasting errors. The MAPE expresses the average error as a percentage of actual patient volume rather than as a raw patient count, which allows comparison of forecast accuracy across EDs of different sizes. Coefficient of determination (*R*^2^) is a score, typically between 0 and 1, describing how well a model’s predictions tracked the actual day-to-day fluctuations in volume: an *R*^2^ of 1.0 would indicate that the model perfectly explained all observed variation in volume, while an *R*^2^ of 0 would indicate that the model explained none of it, performing no better than simply predicting the historical average each day. We also calculated two operational accuracy measures directly relevant to staffing decisions: the proportion of daily forecasts falling within a pre-specified allowable error tolerance, defined as a margin (five, 10, 15, or 20 patients) that an ED might reasonably absorb without meaningfully understaffing or overstaffing for the day.

To assess model stability and temporal variability (error dispersion, as described above), we calculated weekly-level error metrics. For each rolling 7-day forecast window, we computed the MAE across those 7 days, generating a distribution of weekly MAE values across the study period. We summarized this distribution using median values, interquartile ranges ([IQR], defined as the range covering the middle 50% of weekly errors and used here as a measure of week-to-week consistency), mean values, and 95% CIs. This dual approach distinguishes between overall cumulative accuracy (aggregate metrics) and week-to-week reliability (weekly metrics): A model could have a favorable average error while still being unpredictable, alternating between very accurate and very inaccurate weeks, and this second analysis was designed to detect that possibility.

Forecast accuracy differences between modeling approaches were assessed using the Diebold-Mariano (DM) test, a statistical test that determines whether one forecasting method is meaningfully more accurate than another, or whether an observed difference is likely attributable to chance. The XGBoost was designated as the reference model for these comparisons because it achieved the lowest MAPE and RMSE and the highest *R*^2^ among the five models. The DM test compares the loss differential, or difference in forecast errors, between two models using a modified *t*-statistic that accounts for the tendency of forecast errors on nearby days to be correlated with one another. A significance threshold of α = .05 was applied, such that a *P* value < .05 was considered statistically significant; negative DM statistics indicate that the reference model (XGBoost) had smaller errors than the comparison model.

## RESULTS

Table 1 compares two traditional statistical models (SARIMA, ARIMA) with three machine learning models (XGBoost, LGBM, LSTM). As shown in Table 1, median MAE ranged from 7.77 to 8.60 patients per day across SARIMA (7.86), ARIMA (8.60), XGBoost (7.87), and LGBM (7.77), whereas LSTM demonstrated a higher median MAE of 9.59. This indicates that on a typical day, four of the five models missed the actual patient count by approximately 8 patients, while LSTM’s typical miss was closer to 10 patients. Error dispersion, the spread, or variability, of daily errors around that typical value, followed a similar pattern: IQRs of 9.48–10.49 for SARIMA, ARIMA, XGBoost, and LGBM, compared with 11.84 for LSTM. This indicates that LSTM’s errors were both larger on average and more variable from day to day than those of the other four models.

Mean MAE, the average error across all forecasts as opposed to the typical, or median, error above, was lowest for SARIMA (9.60; 95% CI, 9.29–9.91), LGBM (9.64; 95% CI, 9.33–9.94), and XGBoost (9.61; 95% CI, 9.31–9.91). This indicates that these three models missed actual daily volume by approximately 9 to 10 patients per day, on average, and because their CIs overlap substantially, we cannot conclude with confidence that any one of the three clearly outperformed the other two. Mean MAE was higher for ARIMA (10.54; 95% CI, 10.21–10.87) and highest for LSTM (12.01; 95% CI, 11.63–12.39). This same pattern held across every additional metric examined. The RMSE, which penalizes large misses more heavily than MAE, was lowest for XGBoost (12.63), LGBM (12.68), and SARIMA (12.76), followed by ARIMA (13.85) and LSTM (15.83); this indicates that ARIMA, and particularly LSTM, were more prone to occasional large forecasting errors than the other models. The MAPE (error expressed as a percentage of actual volume, allowing comparison independent of ED size) showed a similar ordering, with XGBoost (13.82%), SARIMA (13.82%), and LGBM (13.88%) yielding the lowest relative error, compared with ARIMA (14.93%) and LSTM (17.27%). Model fit, as assessed by *R*^2^ (how well a model’s predictions tracked actual day-to-day changes in volume, on a 0 to 1 scale), was highest for XGBoost (0.5176) and LGBM (0.5144), followed by SARIMA (0.5077); ARIMA showed lower fit (0.4208), and LSTM demonstrated the lowest explanatory performance (0.2404). This indicates that LSTM captured meaningfully less of the true day-to-day variation in ED volume than the other four models.

Table 2 translates these error metrics into a more directly operational question for ED staffing: On what percentage of days would a given model have kept a department’s actual census within a specific, predefined margin (the allowable error tolerance, defined in the methods section) of its forecast? Operational accuracy improved as this allowable error tolerance widened across all approaches. Within 5 patients, SARIMA, ARIMA, XGBoost, and LGBM demonstrated similar performance (31.3–33.3%), whereas LSTM achieved 27.1%. Within 10 patients, SARIMA (60.4%), XGBoost (61.3%), and LGBM (60.4%) exceeded ARIMA (56.9%) and LSTM (51.6%). At 15 patients, coverage increased for XGBoost (79.5%) and LGBM (79.8%), with SARIMA at 80.6%, ARIMA at 75.7%, and LSTM at 70.4%. At 20 patients, a margin most EDs could likely absorb operationally without a major staffing disruption, all non–deep learning models exceeded 87% accuracy (SARIMA 90.3%, ARIMA 87.4%, XGBoost 90.4%, LGBM 90.6%), while LSTM reached 83.3%.

Pairwise comparisons of forecast accuracy using DM testing with XGBoost as the reference are summarized in Table 3. XGBoost demonstrated significantly superior predictive performance compared with both ARIMA (DM = −6.09, *P* < .001) and LSTM (DM = −6.96, *P* < .001). This indicates that XGBoost’s forecasting errors were significantly and meaningfully smaller than those of ARIMA and LSTM, and that this difference is very unlikely to be due to chance. In contrast, no statistically significant difference in predictive accuracy was observed between XGBoost and SARIMA (DM = 0.07, *P* = .94) or between XGBoost and LGBM (DM = −0.52, *P* = .60). This indicates that XGBoost’s errors were statistically indistinguishable from, and not clearly better than, those of SARIMA and LGBM.

Visual inspection of rolling forecasts and residuals supported the quantitative performance metrics. As shown in Figure 1, all models captured long-term trends and seasonal variation in daily ED volumes, including the pronounced decline in early 2020 and subsequent recovery. Tree-based machine learning models (XGBoost and LGBM) and SARIMA demonstrated close alignment with observed volumes across most of the study period, with predictions generally tracking day-to-day variability. The ARIMA and LSTM exhibited greater divergence during periods of abrupt change and high variability.

Scatterplots of predicted vs observed volumes (Figure 2) demonstrated the strongest agreement for XGBoost and LGBM, with dense clustering along the identity (diagonal) line across a broad range of daily volumes. The SARIMA model showed comparable alignment, while ARIMA exhibited increased dispersion at higher volumes. The LSTM predictions were more tightly clustered within a narrower range, reflecting reduced correspondence with observed extremes. These patterns were consistent with the observed differences in *R*^2^ across models.

Residual analyses over time (Figure 3) demonstrated that prediction errors were generally centered around zero for all models, indicating minimal systematic bias. Tree-based models and SARIMA showed relatively stable residual distributions with most errors contained within narrow bounds. The ARIMA and LSTM displayed greater residual dispersion, with more frequent large deviations during periods of heightened variability. Across all approaches, residual magnitude increased during periods of extreme fluctuation in ED volume.

## DISCUSSION

This study compared modern machine learning models against classical time-series approaches for forecasting daily patient volumes in an academic freestanding ED. The SARIMA, XGBoost, and LGBM achieved comparable performance, with absolute mean MAE of 9.60–9.64 patients per day and relative MAPE of 13.82–13.88%. Diebold-Mariano testing revealed that XGBoost significantly outperformed (predicted patient volume better) ARIMA (DM = −6.09, *P* < .001) and LSTM (DM = −6.96, *P* < .001), while showing no significant difference from SARIMA (DM = 0.07, *P* = .94) or LGBM (DM = −0.52, *P* = .60). Weekly error analysis demonstrated overlapping IQR (9.48–10.49) across top-performing models, indicating that performance differences were statistically detectable but operationally modest. In practical terms, all three of these models would have supported similarly reliable weekly staffing decisions over the study period. At clinically relevant thresholds, leading models achieved approximately 80% accuracy within 15 patients and 90% accuracy within 20 patients, supporting practical utility for capacity planning decisions.

The comparable performance of XGBoost and LGBM aligns with previous research demonstrating the effectiveness of gradient boosting methods in healthcare forecasting. Porto and Fogliatto reported XGBoost error rates of 5–14% MAPE in ED forecasting, while our results (13.82% MAPE) reflect inherent variability across different hospital systems and patient populations.^8^ The SARIMA’s competitive performance (13.82% MAPE, identical to XGBoost) underscores that classical statistical methods remain highly effective for ED forecasting, particularly when data exhibit strong seasonal structure.^3^ This finding is consistent with Al Yarubi et al, who similarly found SARIMA to be a strong, interpretable performer relative to other statistical forecasting approaches for ED crowding.^6^ The relatively lower performance of LSTM (MAPE: 17.27%, *R*^2^: 0.24) contrasts with the study by Ming et al, which reported MAPE of 4.87% for LSTM in daily ED arrival forecasting.^13^ This discrepancy may reflect dataset size limitations for deep learning applications or the effectiveness of tree-based methods for capturing temporal patterns in ED volumes.^8^

A key strength of this study is its use of continuous operational data from an ED spanning eight years. Unlike studies that exclude pandemic-related disruptions, retaining these real-world volume fluctuations demonstrates model robustness to external shocks and fills a gap in forecasting research that typically relies on stable, pre-pandemic data. The narrow 95% CIs around mean weekly MAE for top-performing models (9.29–9.91 patients per day) indicate stable forecasting performance throughout this period, enhancing the practical relevance of our findings for EDs that must maintain forecasting capabilities despite unforeseen operational disruptions.

It is also worth clarifying, for ED leaders less familiar with these tools, what would be required to put any of these methods into practice. All five methods evaluated in this study, including the machine learning models, are implemented using free, open-source software rather than proprietary commercial forecasting products; there is no vendor licensing cost inherent to the underlying method itself. In practice, an ED interested in adopting one of these approaches would typically require either an in-house data analytics or biomedical informatics team, or a contracted data scientist or statistician, with access to the department’s own historical daily volume data; this is not a task most ED medical directors would be expected to perform themselves.

However, our results suggest that departments without ready access to machine learning expertise need not be at a disadvantage: SARIMA, a decades-old statistical method that requires substantially less computational infrastructure and specialized expertise than XGBoost, LGBM, or LSTM, performed as well as the best machine learning model in this study. This finding suggests that many EDs could achieve accurate 7-day volume forecasts using simpler, more accessible statistical tools already familiar to hospital biostatistics or quality improvement staff, reserving investment in more complex machine learning infrastructure for settings with additional specific needs (eg, incorporating multiple additional real-time data feeds beyond historical volume alone).

For high-volume EDs where consistent forecast reliability matters for staffing decisions, SARIMA, XGBoost, and LGBM all provide suitable options with comparable week-to-week performance (mean MAE differences of < 0.10 patients per day). Model selection should balance statistical performance with operational priorities: SARIMA offers strong accuracy with high interpretability and low computational burden, while XGBoost and LGBM provide statistically validated but modest performance gains for settings in which incremental improvements justify additional complexity. Although precise point forecasting remains challenging, these models provide high reliability for broader capacity planning decisions such as staffing bands, surge preparedness, and bed allocation.

Future research should focus on external validation across multiple EDs with diverse patient populations and operational characteristics, incorporation of real-time external variables such as weather, local events, and disease surveillance data (an approach for which Hu et al demonstrated meaningful gains over methods relying on historical averages alone^10^), and prospective evaluation of downstream operational outcomes following implementation. Studies should also assess whether operational decisions based on these models equitably affect different patient subgroups.

## LIMITATIONS

This study was conducted using data from a single academic freestanding ED, which may limit generalizability. In accordance with transparent reporting of a multivariable prediction model for individual prognosis or diagnosis, plus (TRIPOD+) AI guidance, a published reporting standard for studies developing prediction models that use AI or machine learning, our analyses represent model development and internal validation without external validation in other hospitals or health systems. Model performance estimates may be optimistic for settings that differ in referral patterns or operational constraints.

## CONCLUSION

Models SARIMA, XGBoost, and LGBM achieved comparable daily emergency department patient volume forecasting performance, demonstrating strong operational reliability for ED capacity planning. Classical time-series methods such as ARIMA and SARIMA remain highly competitive with modern machine learning approaches, offering similar accuracy with greater interpretability. Deep learning (LSTM) performed worse than traditional statistical methods (ARIMA and SARIMA) in this context. For ED medical directors and hospital administrators without dedicated machine learning resources, this finding suggests that adopting a well-established, simpler statistical forecasting method such as SARIMA may represent a practical and sufficient first step toward data-driven staffing decisions. Prospective validation across multiple sites and evaluation of real-world implementation outcomes represent important next steps.

## Figures and Tables

**Figure 1:**
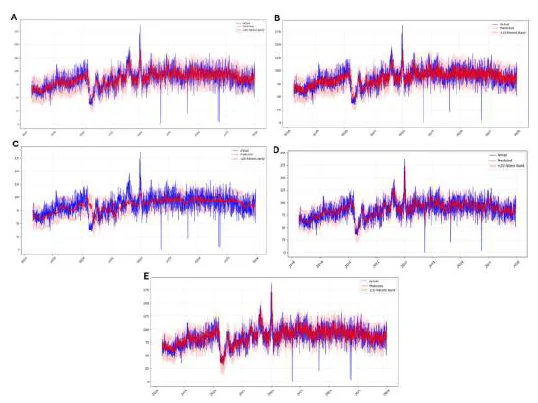
Rolling predictions vs actual emergency department volumes over time. The x-axis represents calendar year, and the y-axis represents daily patient count. In each panel, the blue line represents actual (observed) daily emergency department (ED) patient volume, and the red line represents the model’s forecasted (predicted) volume for that same day. The lightly shaded red region surrounding the red line represents a 20-patient band around the prediction, a visual reference for the operational accuracy threshold reported in Table 2. Where the blue line extends above the red, the model under-predicted patient volume for that day; where the blue line falls below the red, the model over-predicted volume. Panels show model-specific predictions versus actual volumes: (**A**) eXtreme gradient boosting, (**B**) light gradient boosting machine (**C**) long short-term memory (**D**) autoregressive integrated moving average, and (**E**) seasonal autoregressive integrated moving average, in a study comparing five forecasting approaches using an 8-year dataset from an academic freestanding ED.

**Figure 2:**
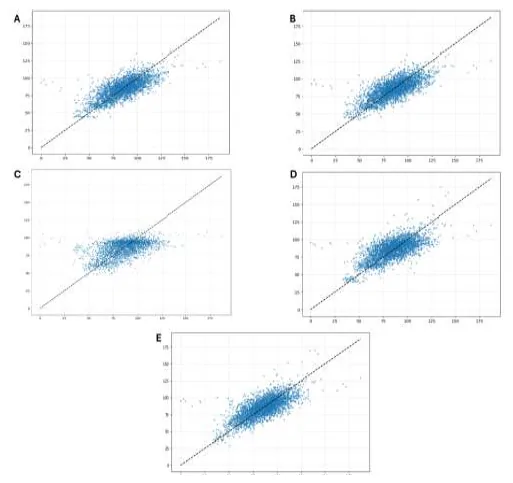
Scatterplots of rolling predicted versus observed daily emergency department (ED) patient volumes across modeling approaches in a study of five forecasting approaches using an 8-year dataset from an academic freestanding ED. The x-axis represents actual patient volume, and the y-axis represents predicted patient volume. The diagonal line in each panel represents perfect prediction: if a model predicted patient volume perfectly, every blue dot would fall exactly on this line. Each blue dot represents one day’s forecast; the vertical distance of a dot from the diagonal line reflects how much that model’s prediction missed the actual patient volume for that day. Although the five panels appear visually similar at first glance, the coefficient of determination (*R*^2^) reported for each panel, which reflects how tightly the dots cluster around the diagonal line overall, shows that XGBoost (**A**) and LGBM (**B**) achieved the closest agreement with actual volumes, followed by SARIMA (**E**) and ARIMA (**D**), while LSTM (**C**) showed the most scatter away from the diagonal, consistent with its higher error metrics in Table 1. (**A**) eXtreme gradient boosting (*R*^2^ = 0.5176), (**B**) light gradient boosting machine (*R*^2^ = 0.5144), (−) long short-term memory (*R*^2^ = 0.2404), (**D**) autoregressive integrated moving average (R^2^ = 0.4208), and (**E**) seasonal autoregressive integrated moving average (*R*^2^ = 0.5077).

**Figure 3:**
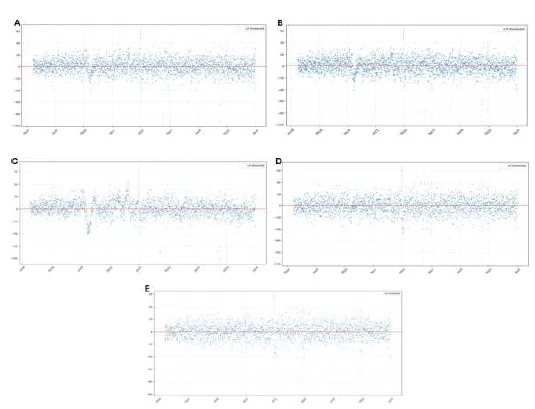
Temporal distribution of rolling forecast residuals over time across predictive models for daily emergency department patient volumes. Residuals are defined as the difference between predicted and observed values. A residual near zero indicates an accurate day’s forecast, while a residual farther from zero indicates a day the model missed by a larger margin, with the sign of the residual indicating the direction of the error (over-prediction or under-prediction). The x-axis represents calendar year, and the y-axis represents patient prediction error. Panels correspond to (**A**) eXtreme gradient boosting, (**B**) light gradient boosting machine (**C**) long short-term memory (**D**) autoregressive integrated moving average, and (**E**) seasonal autoregressive integrated moving average.

**Table 1:** Summary of predictive performance metrics for models forecasting daily emergency department (ED) arrival volume, grouped by model type (traditional statistical vs machine learning) in a study of five forecasting approaches using an 8-year dataset from an academic freestanding EDs.

Model	Model Type	Median MAE	IQR	Mean MAE	95% CI Lower	95% CI Upper	RMSE	MAPE (%)	R^2^
**SARIMA**	*Traditional statistical*	7.86	9.48	9.60	9.29	9.91	12.76	13.82	0.5077
**ARIMA**	*Traditional statistical*	8.60	10.49	10.54	10.21	10.87	13.85	14.93	0.4208
**XGBoost**	*Machine learning*	7.87	9.74	9.61	9.31	9.91	12.63	13.82	0.5176
**LGBM**	*Machine learning*	7.77	9.89	9.64	9.33	9.94	12.68	13.88	0.5144
**LSTM**	*Machine learning*	9.59	11.84	12.01	11.63	12.39	15.83	17.27	0.2404

*ARIMA*, autoregressive integrated moving average; *IQR*, interquartile range; *LGBM*, Light Gradient Boosting Machine; *LSTM*, long short-term memory; *MAE*, mean absolute error; *MAPE*, mean absolute percentage error; *RMSE*, root mean squared error; *SARIMA*, seasonal autoregressive integrated moving average; *XGBoost*, eXtreme Gradient Boosting.

**Table 2:** Operational accuracy of forecasting models in a study of five forecasting approaches using an 8-year dataset from an academic freestanding emergency department.

Model	Model Type	Within 5 Patients (%)	Within 10 Patients (%)	Within 15 Patients (%)	Within 20 Patients (%)
**SARIMA**	*Traditional statistical*	33.3	60.4	80.6	90.3
**ARIMA**	*Traditional statistical*	31.3	56.9	75.7	87.4
**XGBoost**	*Machine learning*	33.3	61.3	79.5	90.4
**LGBM**	*Machine learning*	33.1	60.4	79.8	90.6
**LSTM**	*Machine learning*	27.1	51.6	70.4	83.3

*ARIMA*, autoregressive integrated moving average; *LGBM*, Light Gradient Boosting Machine; *LSTM*, long short-term memory; *SARIMA*, seasonal autoregressive integrated moving average; *XGBoost*, eXtreme Gradient Boosting.

**Table 3:** Pairwise Diebold-Mariano test results with eXtreme gradient boosting as reference in a study of five forecasting approaches using an 8-year dataset from an academic freestanding emergency department.

Comparison (vs XGBoost reference)	DM Statistic	*P* Value
XGBoost vs SARIMA (traditional statistical)	0.07	.94
XGBoost vs ARIMA (traditional statistical)	−6.09	< .001
XGBoost vs LGBM (machine learning)	−0.52	.60
XGBoost vs LSTM (machine learning)	−6.96	< .001

*ARIMA*, autoregressive integrated moving average; *DM*, Diebold-Mariano; *LGBM*, Light Gradient Boosting Machine; *LSTM*, long short-term memory; *SARIMA*, seasonal autoregressive integrated moving average; *XGBoost*, eXtreme Gradient Boosting.
